# Adipose Tissue Regulates Pulmonary Pathology during TB Infection

**DOI:** 10.1128/mBio.02771-18

**Published:** 2019-04-16

**Authors:** Janeesh Plakkal Ayyappan, Usha Ganapathi, Kezia Lizardo, Christopher Vinnard, Selvakumar Subbian, David S. Perlin, Jyothi F. Nagajyothi

**Affiliations:** a Public Health Research Institute, New Jersey Medical School, Rutgers Biomedical and Health Sciences, Newark, New Jersey, USA; Washington University in St. Louis School of Medicine

**Keywords:** adipose tissue, fat loss, Mycobacterium tuberculosis, TB reactivation, foamy macrophages, wasting

## Abstract

Tuberculosis (TB), caused by Mycobacterium tuberculosis infection, remains a major cause of mortality and morbidity worldwide. One-third of the world population is infected with M. tuberculosis, and about 15 million people with latent tuberculosis infection (LTBI) reside in the United States. An estimated 10% of individuals with LTBI are at risk of progressing to active disease. Loss of body mass, or wasting, accompanied by a significant reduction of body fat is often associated with active TB disease and is considered to be immunosuppressive and a major determinant of severity and outcome of disease. While the lungs are the primary site of M. tuberculosis infection and TB manifestation, recent reports have shown that adipose tissue serves as an important reservoir for M. tuberculosis. In this article, we investigated the association between M. tuberculosis infection, adipose tissue, and TB disease progression using a transgenic inducible “fatless” model system, the FAT-ATTAC (fat apoptosis through targeted activation of caspase 8) mouse. By selectively ablating fat tissue during M. tuberculosis infection, we directly tested the role of fat cell loss and adipose tissue physiology in regulating pulmonary pathology, bacterial burden, and immune status. Our results confirm the presence of M. tuberculosis in fat tissue after aerosol infection of mice and show that loss of fat cells is associated with an increase in pulmonary M. tuberculosis burden and pathology. We conclude that acute loss of adipose tissue during LTBI may predispose the host to active TB disease.

## Abstract (precis)

Although the lungs are the port of entry and the predominant site of TB disease manifestation, we and others have demonstrated that M. tuberculosis also persists in adipose tissue of aerosol-infected animals and directly or indirectly alters adipose tissue physiology, which in turn alters whole-body immuno-metabolic homeostasis. Our present report demonstrates a direct effect of loss of adipocytes (fat cells) on promoting the severity of pulmonary pathogenesis during TB, advancing our understanding of the pathogenic interactions between wasting and TB activation/reactivation.

## INTRODUCTION

Tuberculosis (TB) is a major global health problem caused by the bacterium Mycobacterium tuberculosis. TB continues to be a major cause of morbidity and mortality, primarily in low- and middle-income countries. According to CDC data, in 2017 there were 10.4 million new cases of TB and 1.7 million deaths worldwide (1). It is well documented that progressive loss of body mass, or wasting, is associated with active TB disease (2). However, it is not fully understood whether active TB disease causes the weight loss due to lack of appetite and malabsorption of nutrients or whether, conversely, malnutrition promotes TB activation. Wasting is a prominent feature of TB and one of the factors most closely associated with disease severity and the outcome of treatment (3). Wasting in active TB patients results from a loss of both nonfat and fat masses, and the loss of body fat mass is observed mainly in the trunk and limbs (3, 4). Our recently published study of a rabbit TB model suggested that body fat mass may regulate pulmonary TB pathogenesis (5). In this study, we explore the association between acute fat loss and pulmonary TB pathogenesis and investigate fat-tissue-associated risk factors in the pathogenesis of TB.

Fat (adipose) tissue is a complex, essential, and highly active metabolic and endocrine organ that constitutes 15 to 25% of total body mass. Besides adipocytes, adipose tissue includes a connective tissue matrix, nerve tissue, stromal vascular cells, and immune cells. About two-thirds of the stromal vascular fraction of adipose tissue is comprised of immune cells (e.g., macrophages, NKT cells, mast cells, neutrophils, and T and B lymphocytes), which contribute to whole-body immune signaling via secreted adipokines and cytokines such as adiponectin, leptin, tumor necrosis factor alpha (TNF-α), gamma interferon (IFN-γ), interleukin-6 (IL-6), and IL-10 (6). Previously, we reported an association between key adipokine levels and the dynamics of M. tuberculosis pathogenesis in the lungs and adipose tissue using a rabbit model of pulmonary infection, with two clinical M. tuberculosis isolates that produced divergent outcomes of disease progression (5). We showed that the expression of the adipose tissue-specific protein adiponectin, an anti-inflammatory adipokine, was decreased in adipose tissue and increased in the lungs of M. tuberculosis-infected rabbits, suggesting a possible association between adipose tissue physiology and lung pathology (5). Although the lungs serve as the port of entry and the predominant site of TB disease manifestation, recent reports have also shown that adipose tissue serves as an important niche and reservoir for M. tuberculosis and that M. tuberculosis infection alters adipose tissue biology (7–9). Yet, the role of adipose tissue in the progression and pathology of M. tuberculosis infection has remained unclear.

In the present study, we have investigated whether adipose tissue and acute adipocyte loss plays a regulatory role in pulmonary pathology during M. tuberculosis infection, using a transgenic inducible “fatless” model system, the FAT-ATTAC (fat apoptosis through targeted activation of caspase 8) mouse (10, 11). As demonstrated previously, FAT-ATTAC mice are indistinguishable from their wild-type littermates. However, apoptosis of adipocytes can be induced at any developmental stage by administration of an FK1012 analog, leading to the dimerization of a membrane-bound, adipocyte-specific caspase 8-FK506 binding protein (FKBP) fusion. Within 2 weeks of the dimerizer administration, FAT-ATTAC mice show severely reduced (>95%) levels of circulating adipokines and substantially reduced levels of adipose tissue (10, 11). This model has allowed us to directly test the role of adipocytes and adipose tissue physiology in regulating pulmonary pathology, bacterial burden, and immune status by selectively ablating fat tissue during M. tuberculosis infection. Our results show that M. tuberculosis is present in fat tissue after aerosol infection of mice and that acute loss of fat cells is associated with an increase in the pulmonary M. tuberculosis burden. We also demonstrate that acute fat loss enhances the levels of pulmonary macrophages but promotes an anti-inflammatory environment in the lungs of M. tuberculosis*-*infected mice. This study supports an emerging view that adipose tissue pathophysiology plays a major role in wasting and, as a consequence, determining the activation and reactivation of TB infection.

## RESULTS

### M. tuberculosis persists in visceral adipose tissue and alters adipose tissue morphology.

**FIG 1:**
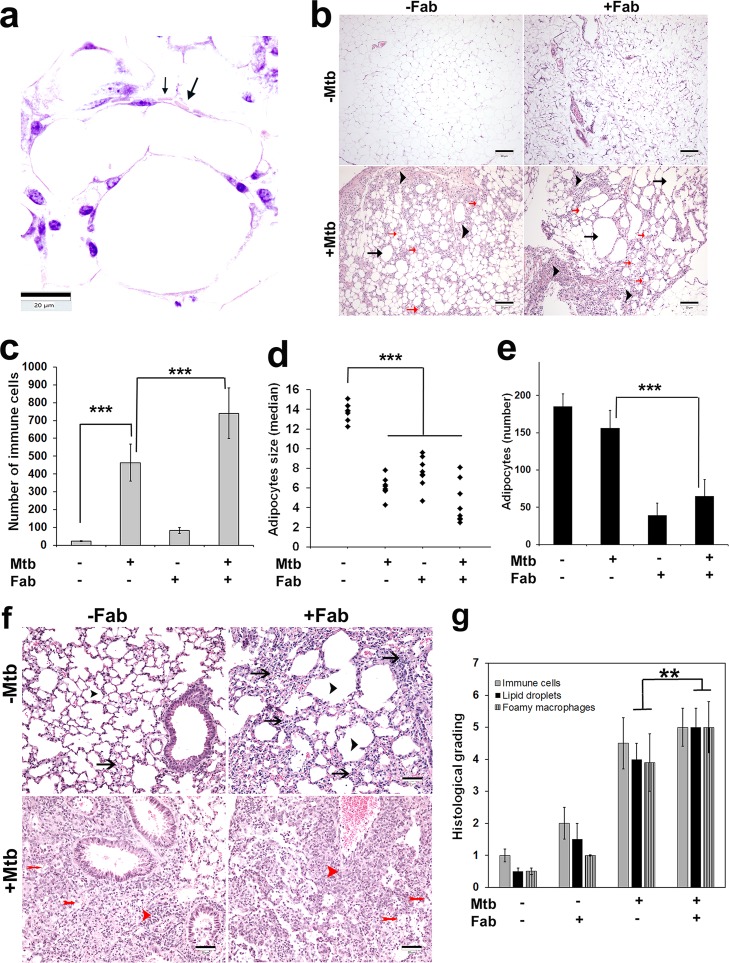
M. tuberculosis persists in adipose tissue, and fat ablation increases pulmonary pathology in M. tuberculosis-infected FAT-ATTAC mice. (a) M. tuberculosis is present in the visceral fat sections of FAT-ATTAC mice aerosol infected with H37Rv as demonstrated by Ziehl-Neelsen (ZN) staining (black arrows) at 30 dpi (×60 magnification). Scale bar = 20 μm. (b) M. tuberculosis infection alters adipose tissue pathology by causing adipocyte hypertrophy (black arrows, hypertrophied adipocytes), infiltration of immune cells into adipose tissue (black arrowheads), and loss of adipocytes (red arrows, smaller adipocytes) in FAT-ATTAC mice aerosol infected with H37Rv, as shown by H&E staining (×10 magnification) at 30 dpi for fat-ablated mice (Fab). *n* = 5. Scale bar = 20 μm. (c to e) Analysis of infiltrated immune cells (c), size of adipocytes (d), and number of adipocytes (e) in adipose tissue during M. tuberculosis infection and/or fat ablation as quantitated by counting the number of immune cells/adipocytes and measuring manually the size of adipocytes of H&E-stained adipose tissue at 5 images per section in each group (×40 images were used). The values plotted are mean (a and e) or median (d) ± standard deviation (SD) from *n* = 5. ***, *P* < 0.01. (f) H&E analysis of the lungs showed increased alveolar space (decreased number) (black arrowheads) and septal thickness (black arrows) in uninfected fat-ablated mice compared to uninfected control mice (top panel). Histological analysis of the lungs demonstrated increased lung pathology (infiltrated immune cells [red arrowheads] and foamy macrophages [red double arrows]) in M. tuberculosis-infected mice compared to uninfected mice as demonstrated by H&E staining. Scale bar = 20 μm. (g) Histological grading of lung pathology was carried out according to experimental groups and classified in terms of infiltrated immune cells and presence of foamy macrophages (Fig. S2). Each class was graded on a 5-point scale ranging from 0 to 5+ as discussed in Materials and Methods. Values plotted are mean ± standard error (SE) from *n* = 5.The error bars represent the standard error of the mean. *, *P* ≤ 0.05, **, *P* ≤ 0.01, and ***, *P* ≤ 0.001, compared to uninfected fat-unablated mice.

Previously, we and others have shown that M. tuberculosis infects cultured 3T3L1 adipocytes and alters adipokine levels (5, 12, 13). Here, we examined whether M. tuberculosis resides in the adipose tissue of aerosol-infected mice. We infected FAT-ATTAC mice with the M. tuberculosis H37Rv strain and examined sections of visceral fat pads for the presence of M. tuberculosis. Ziehl-Neelsen (ZN) staining of the sections demonstrated the presence of acid-fast bacilli in the vicinity of lipid droplets in M. tuberculosis*-*infected mice at 30 days postinfection (dpi) (Fig. 1a). The morphology of the adipose depots was altered during acute M. tuberculosis infection (Fig. 1b), and quantitative analysis of hematoxylin and eosin (H&E)-stained adipose tissue sections revealed significantly increased immune cell infiltration and decreased size and number of lipid droplets in the infected mice compared to uninfected mice at 30 dpi (Fig. 1c to e). Even though the numbers of adipocyte cells were not significantly different between the uninfected and infected groups, the sizes of adipocytes were significantly decreased in infected mice compared to uninfected mice (Fig. 1d and e). Some of the adipocytes were hypertrophied and surrounded by infiltrated immune cells.

10.1128/mBio.02771-18.2 FIG S2 Histological analysis demonstrated a significant increase in the levels of pulmonary lipid droplets in fat-ablated (+Fat) mice compared to fat-unablated (−Fat) mice during M. tuberculosis infection (*n* = 5). (a) H&E analysis of the pulmonary sections demonstrated increased accumulation of the lipid droplets (arrow) and presence of foamy macrophages (red arrowheads) in the lungs of infected fat-ablated mice (×60 magnification). (b) ZN-stained lung sections demonstrate the presence of M. tuberculosis (black arrows) and cholesterol crystals in infected fat-ablated mice compared to infected fat-unablated mice. (c) Immunohistochemical staining of lung tissues displays elevated levels of perilipin (open arrowheads) in fat-ablated mice compared to fat-unablated mice at 30 dpi. Download FIG S2, TIF file, 2.8 MB. Copyright © 2019 Ayyappan et al. 2019 Ayyappan et al. This content is distributed under the terms of the Creative Commons Attribution 4.0 International license.

### Loss of adipose tissue exacerbates lung histopathology in a murine TB model.

Histological analysis of H&E-stained lung sections demonstrated worsened lung pathology in M. tuberculosis-infected mice compared to uninfected mice, which included decreased alveolar air space and increased septal thickening, as well as increased levels of lipid droplets and infiltrated immune cells such as macrophages, neutrophils, T cells, and mast cells (Fig. 1f and g; see Fig. S2a in the supplemental material). H&E-stained sections of M. tuberculosis-infected lungs also showed the presence of foamy macrophages (Fig. 1f and g; Fig. S2a). The levels of infiltrated immune cells and lipid droplets were even further elevated in M. tuberculosis-infected mice that underwent fat ablation compared to infected fat-unablated mice, suggesting that loss of adipose tissue enhances pulmonary pathology during M. tuberculosis infection (Fig. 1f and g and Fig. S2a and b). H&E-stained sections also demonstrated the presence of higher levels of foamy macrophages in the lungs of M. tuberculosis-infected fat-ablated mice compared to infected fat-unablated mice (Fig. 1f and g; Fig. S2a). Also, fat-ablated uninfected mice displayed enlarged alveolar air spaces and increased septal thickening in the lungs compared to control mice (uninfected fat-unablated mice) (Fig. 1f and g). ZN-stained lung sections demonstrated the presence of increased levels of cholesterol crystals in infected fat-ablated mice compared to infected fat-unablated mice (Fig. S2b). Immunohistochemical analysis of perilipin staining demonstrated a significant increase in the levels of lipid droplets in the lung sections of infected fat-ablated mice compared to infected fat-unablated mice (Fig. S2c), consistent with exacerbated lung pathology.

### Fat ablation increases M. tuberculosis load in the lungs of infected mice.

**FIG 2:**
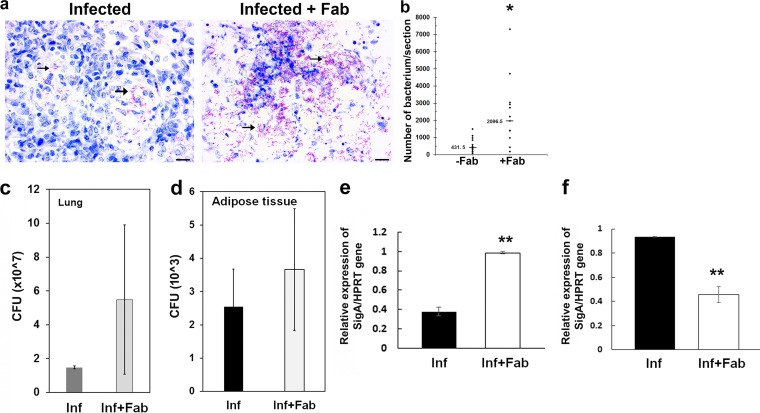
Fat ablation increases bacterial load in the lungs demonstrated by ZN staining, CFU counts, and qPCR analysis. (a) Fat ablation (Fab [right panel]) during acute M. tuberculosis infection in FAT-ATTAC mice (*n* = 10) show significantly increased pulmonary bacterial loads (arrows) compared to fat-unablated mice (left) as demonstrated by ZN staining of lung section from the respective group (scale bar = 20 μm). (b) Quantitative analysis of bacterial load in ZN images (4 sections/mouse; magnification, ×40) determined by manually counting the stained bacteria and represented as a scatter graph. The median values of bacterial load estimated were 431.5 and 2,096.5 (*P* ≤ 0.01) between M. tuberculosis-infected, fat-unablated (−Fab) and fat-ablated (+Fab) animals. *, *P* ≤ 0.05. (c) Number of CFU/gram in the lungs of M. tuberculosis*-*infected fat-unablated (Inf) and infected fat-ablated (Inf+Fab) mice at 30 dpi. Bacterial counts were determined from lung homogenates plated on agar medium. The values plotted are mean ± SE from *n* = 5 animals per group. (d) Number of CFU/0.5 g in the visceral adipose tissues of M. tuberculosis-infected fat-unablated (Inf) and infected fat-ablated (Inf+Fab) mice at 30 dpi. Bacterial counts were determined from adipose tissue homogenates plated on agar medium. Values plotted are mean ± SE from *n* = 5 animals per group. (e) qPCR analysis demonstrated a significant increase in the levels of M. tuberculosis *sigA* gene expression in the lungs of M. tuberculosis-infected fat-ablated (Inf+Fab) mice compared to infected fat-unablated (Inf) mice at 30 dpi. The expression level of the housekeeping HPRT gene was used as a control. The values plotted are mean ± SE from *n* = 5 animals per group. **, *P* ≤ 0.01. (f) qPCR analysis demonstrated a significant decrease in the levels of M. tuberculosis *sigA* genes in the adipose tissues of M. tuberculosis-infected fat-ablated (Inf+Fab) mice compared to infected fat-unablated (Inf) mice at 30 dpi. The expression level of the housekeeping HPRT gene was used as a control. The values plotted are mean ± SE from *n* = 5 animals per group. **, *P* ≤ 0.01.

Bacterial loads in the lung sections of M. tuberculosis aerosol-infected mice were evaluated, as detected by ZN staining (Fig. 2a). To determine how fat ablation affects pulmonary bacterial loads, we performed blinded quantitative analyses of bacterial loads in the ZN images. Sections of the lungs of M. tuberculosis-infected fat-ablated mice contained a significantly higher load (≈5-fold) of bacteria compared to fat-unablated infected mice (Fig. 2b). Although a parallel assessment of lung bacterial loads by CFU analysis also showed higher CFU levels in the lungs of fat-ablated mice compared to fat-unablated mice at 30 dpi (Fig. 2c), this difference was not statistically significant. Analysis of CFU in the visceral fat tissues similarly revealed no significant difference between the infected fat-unablated and infected fat-ablated mice (Fig. 2d). The mRNA levels of the M. tuberculosis *sigA* gene were significantly higher in the lungs of infected fat-ablated mice compared to infected fat-unablated mice (Fig. 2e). The visceral fat deposits of M. tuberculosis-infected animals also contained *sigA* RNAs; however, the levels of *sigA* transcripts were significantly lower (*P* ≤ 0.01) in the adipose tissue of infected fat-ablated mice compared to infected fat-unablated mice (Fig. 2f). Overall, these data indicate that fat ablation resulted in an increased bacterial load in the lungs.

We also analyzed the growth status of M. tuberculosis (i.e., actively replicating or dormant) in the lungs and visceral fat tissues of M. tuberculosis-infected mice with or without fat ablation by measuring the relative expression of M. tuberculosis genes associated with active replication (*esat6* and *agf85a*) or dormancy (*acr* and *dosR*). Quantitative PCR (qPCR) analysis demonstrated a significant increase in both replication and dormancy genes in the lungs of infected fat-ablated mice compared to infected fat-unablated mice at 30 dpi (see Fig. S3a in the supplemental material). In contrast, the expression levels of both sets of genes were significantly decreased in the visceral fat of infected fat-ablated mice compared to infected fat-unablated mice at 30 dpi (Fig. S3b).

10.1128/mBio.02771-18.3 FIG S3 qPCR analysis demonstrated differentially altered expression of M. tuberculosis genes in the lungs and adipose tissues of infected mice that underwent fat ablation during M. tuberculosis infection (*n* = 5). (a) The levels of M. tuberculosis genes associated with active replication (*esat6* and *agf85A*) and dormancy (*acr* and *dosR*) significantly increased in the lungs of infected fat-ablated (Inf+Fab) mice compared to infected fat-unablated (Inf) mice at 30 dpi. (b) The levels of M. tuberculosis genes associated with active replication (*esat6* and *agf85A*) and dormancy (*acr* and *dosR*) significantly decreased in the adipose tissues of infected fat-ablated (Inf+Fab) mice compared to infected fat-unablated (Inf) mice at 30 dpi. The error bars represent standard error of the mean. *, *P* ≤ 0.05, **, *P* ≤ 0.01, and ***, *P* ≤ 0.001, compared to uninfected fat-unablated mice. #, *P* ≤ 0.05, ##, *P* ≤ 0.01, and ###, *P* ≤ 0.001, compared to infected untreated mice. Inf, M. tuberculosis infected; Fab, fat ablated. Download FIG S3, TIF file, 1.4 MB. Copyright © 2019 Ayyappan et al. 2019 Ayyappan et al. This content is distributed under the terms of the Creative Commons Attribution 4.0 International license.

### M. tuberculosis infection causes loss of body weight and fat mass.

Body weight analysis showed that fat ablation caused a significant decrease in the weights of uninfected fat-ablated mice compared to uninfected fat-unablated mice (see Fig. S4a and S4b in the supplemental material). There was a statistically significant ∼3% decrease in the weights of M. tuberculosis-infected mice (both fat ablated and unablated: *P* ≤ 0.05 and *P* ≤ 0.005, respectively) compared to uninfected mice at 30 dpi. Although M. tuberculosis infection caused a reduction in body weight at 30 dpi, we had expected a significant decrease in the body weights of infected fat-ablated mice compared to uninfected fat-ablated mice due to both infection and fat ablation. Instead, the body weights of infected fat-ablated mice were significantly higher (*P* ≤ 0.005) than those of uninfected fat-ablated mice. We analyzed changes in visceral fat mass during infection and fat ablation (Fig. S4c) and found a significant reduction in visceral fat due both to infection and fat ablation in the infected fat-ablated mice compared to all other groups (Fig. S4c). We speculate that even though infected fat-ablated mice were losing greater levels of body fat during infection, they may have been suffering increased accumulation of fluid caused by inflammation (edema), which could contribute to the body weight.

10.1128/mBio.02771-18.4 FIG S4 Body weight measurements and visceral fat loss in mice during M. tuberculosis infection and fat ablation. (a) Percentage change in the body weights of M. tuberculosis-infected and fat-ablated/unablated mice at 30 dpi compared to 0 dpi. The values plotted are mean ± SE of *n* = 5. (b) Body weights of each mouse of different experimental groups at 30 dpi presented as a scatter graph (horizontal bars indicate median weight). (c) Representative pictures of a dissected mouse from each of the different experimental groups displaying change in visceral fat (red arrow) mass during infection and fat ablation at 30 dpi. Download FIG S4, TIF file, 2.4 MB. Copyright © 2019 Ayyappan et al. 2019 Ayyappan et al. This content is distributed under the terms of the Creative Commons Attribution 4.0 International license.

### Fat ablation increases the levels of adipogenic markers in the lungs of M. tuberculosis-infected mice.

**FIG 3:**
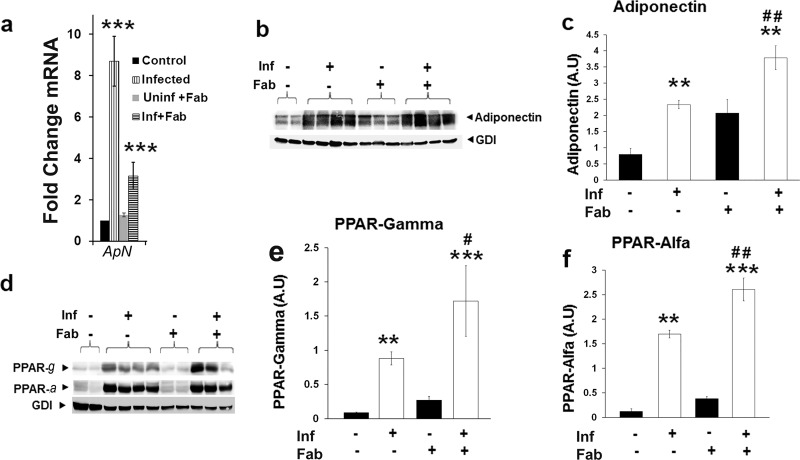
Fat ablation elevates the levels of adipogenic markers and lipid droplets in the lungs of M. tuberculosis-infected mice at 30 dpi. (a) qPCR analysis of mRNA levels of adiponectin (ApN) in the lungs of M. tuberculosis-infected and uninfected mice with (Fab) and without fat ablation normalized to HPRT mRNA. (b) Immunoblot analysis of adiponectin (high molecular weight) in the lung lysates of infected and uninfected mice with and without fat ablation. GDI was used as loading control. (c) Fold changes in the protein levels of adiponectin were normalized to GDI expression and are represented as a bar graph. (d) Immunoblot analysis of PPARs (PPAR-γ and -α) in the lung lysates of infected and uninfected mice with and without fat ablation. GDI was used as loading control. (e) Fold changes in the protein levels of PPAR-γ were normalized to GDI expression and are represented as a bar graph. (f) Fold changes in the protein levels of PPAR-α were normalized to GDI expression and are represented as a bar graph. The error bars represent standard error of the mean. *, *P* ≤ 0.05, **, *P* ≤ 0.01, and ***, *P* ≤ 0.001, compared to uninfected fat-unablated mice. #, *P* ≤ 0.05, ##, *P* ≤ 0.01, and ###, *P* ≤ 0.001, compared to infected untreated mice. Inf, M. tuberculosis infected; Fab, fat ablated.

Previously we demonstrated a significant increase in the levels of pulmonary adiponectin and upregulation of the adiponectin gene in the lungs of M. tuberculosis-infected rabbits (5). In the present study, we observed similar results in a murine TB model. qPCR analysis demonstrated a significant increase in adiponectin mRNA levels in the lungs of infected mice (9- and 3-fold increases in fat-ablated and unablated mice, respectively) compared to uninfected mice (Fig. 3a). Immunoblot analysis demonstrated a significant increase (3.4-fold) in the levels of adiponectin in the lungs of M. tuberculosis*-*infected mice compared to uninfected mice at 30 dpi (Fig. 3b and c). Since adiponectin is known to regulate both adipogenesis and lipid oxidation (13, 14), we analyzed the levels of PPAR-γ (peroxisome proliferator-activated receptor gamma) and PPAR-α, key regulators of adipogenesis and lipid oxidation, respectively, by Western blotting. We found that M. tuberculosis infection significantly increased (>5- and 8-fold, respectively) the levels of both PPAR-γ and PPAR-α (Fig. 3d and e) in the lungs at 30 dpi. Fat ablation further increased the protein levels of adiponectin, PPAR-γ, and PPAR-α in the lungs of M. tuberculosis-infected mice compared to fat-unablated mice (Fig. 3b to e), consistent with the exacerbated lung pathology.

### M. tuberculosis infection activates pulmonary lipases, and loss of body fat further enhances their activity.

**FIG 4:**
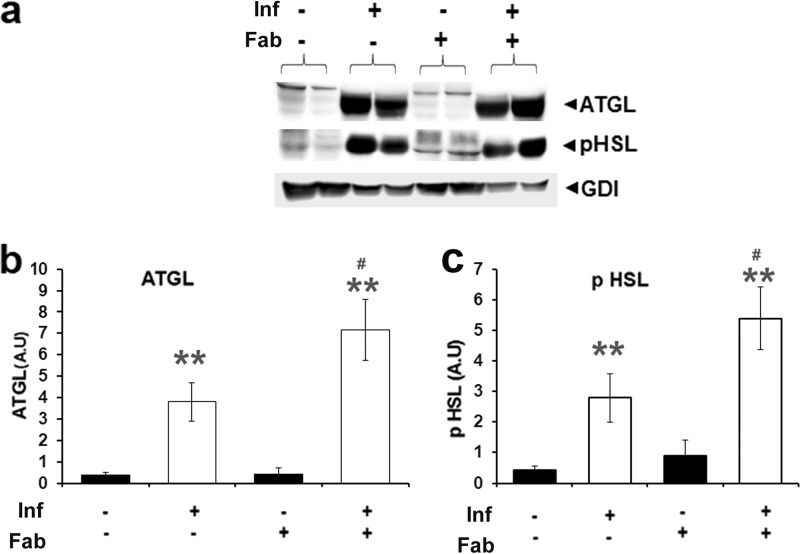
Fat ablation elevates the levels of lipases in the lungs of M. tuberculosis-infected mice at 30 dpi. (a) Immunoblot analysis of lipases (ATGL and p-HSL) in the lung lysates of infected and uninfected mice with and without fat ablation. GDI was used as a loading control. (b) Fold changes in the protein levels of ATGL were normalized to GDI expression and are represented as a bar graph. (c) Fold changes in the protein levels of p-HSL were normalized to GDI expression and are represented as a bar graph. The error bars represent standard error of the mean. *, *P* ≤ 0.05, **, *P* ≤ 0.01, and ***, *P* ≤ 0.001, compared to uninfected fat-unablated mice. #, *P* ≤ 0.05, ##, *P* ≤ 0.01, and ###, *P* ≤ 0.001, compared to infected untreated mice. Inf, M. tuberculosis infected; Fab, fat ablated.

Having observed increased numbers of lipid droplets in the lungs of M. tuberculosis-infected mice, we determined the levels of pulmonary lipases involved in degradation of lipid droplets. Adipose triglyceride lipase (ATGL) and hormone-sensitive lipase (HSL) are involved in intracellular degradation of triacylglycerols to diacylglycerol (by ATGL and HSL) and then to monoacylglycerols (by HSL). Western blot analyses revealed significantly increased levels of ATGL (9.5-fold) and phosphorylated HSL (p-HSL [5.4-fold]) in the lungs of infected mice compared to uninfected mice (Fig. 4a to c). Levels of lipases were further elevated (*P* ≤ 0.05) in the lungs of infected fat-ablated mice compared to infected fat-unablated mice (Fig. 4a to c). Thus, M. tuberculosis infection induces the expression and activity of pulmonary lipases, and this induction is enhanced further upon adipose tissue loss.

### Loss of adipose tissue increases pulmonary macrophage levels and alters inflammatory signaling.

**FIG 5:**
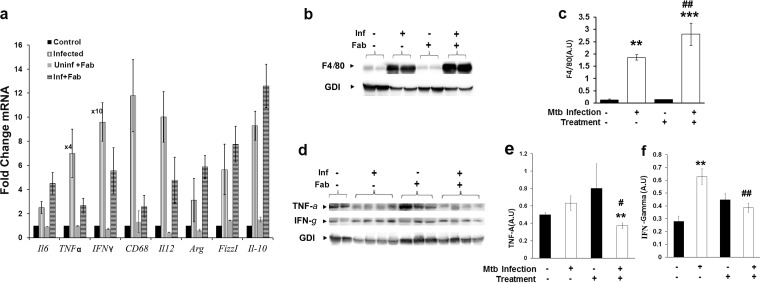
Loss of adipose tissue increases pulmonary M2 macrophage levels and alters inflammatory signaling at 30 dpi. (a) qPCR analysis of mRNA levels of various proinflammatory markers (IL-6, TNF-α, IFN-γ, CD68, and IL-12) and anti-inflammatory cytokines (IL-10), as well as the markers of M2 macrophages (Arg1 and FizzI) in the lungs of M. tuberculosis-infected (fat-unablated and -ablated) mice. Fold changes in the mRNA levels of inflammatory genes were compared to the levels in uninfected control mice and normalized to HPRT gene expression. (Fold increases in TNF-α and IFN-γ were 4 and 10 times higher, respectively, than the represented *y* axis in infected fat-unablated mice.) (b) Immunoblot analysis of F4/80 as a surrogate for macrophage levels in the lung lysates of infected and uninfected mice with and without fat ablation using anti-F4/80 antibody. GDI was used as a loading control. (c) Fold changes in the protein levels of F4/80 were normalized to GDI expression and are represented as bar graph. (d) Immunoblot analysis of proinflammatory cytokines (TNF-α and IFN-γ) in the lungs of M. tuberculosis-infected and uninfected mice with and without fat ablation. GDI was used as a loading control. (e) Fold changes in the protein levels of TNF-α were normalized to GDI expression and are represented as a bar graph. (f) Fold changes in the protein levels of IFN-γ were normalized to GDI expression and are represented as a bar graph. The error bars represent standard error of the mean. *, *P* ≤ 0.05, **, *P* ≤ 0.01, and ***, *P* ≤ 0.001, compared to uninfected fat-unablated mice. #, *P* ≤ 0.05, ##, *P* ≤ 0.01, and ###, *P* ≤ 0.001, compared to infected untreated mice. Inf, M. tuberculosis infected; Fab, fat ablated.

Histology analysis of lung sections demonstrated a significant increase in the levels of infiltrating immune cells in M. tuberculosis-infected mice (Fig. 1f and g). Macrophages derived from infiltrated monocytes undergo specific differentiation to the M1 or M2 phenotype, depending on the local tissue environment. M1 macrophages are classically activated and produce proinflammatory cytokines, nitric oxide (NO), or reactive oxygen intermediates (ROI) to protect against bacteria and viruses. In contrast, M2 macrophages are mainly associated with wound healing and tissue repair. To analyze the inflammatory status of the lungs during infection and after fat ablation, we performed qPCR analysis and measured the levels of various proinflammatory (IL-6, TNF-α, IFN-γ, CD68, and IL-12) and anti-inflammatory (IL-10) cytokines, and the mRNA levels of M2 macrophage markers (Arg1 and FizzI) (Fig. 5a). The mRNA levels of all these markers were significantly increased (*P* ≤ 0.01 to 0.001) in the lungs of M. tuberculosis-infected (both fat-ablated and -unablated) mice compared to uninfected control mice. Comparing the lungs of M. tuberculosis-infected fat-ablated mice to infected fat-unablated mice, we found that the mRNA levels of most of the proinflammatory cytokines and markers were decreased (TNF-α, 10.56-fold; IFN-γ, 1.7-fold; CD68, 4.5-fold; and IL-12, 2.5-fold) and the mRNA levels of anti-inflammatory cytokines IL-10 and Arg1 were increased (1.4- and 2-fold, respectively; *P* ≤ 0.01). We also performed quantitative protein analysis by immunoblotting to measure macrophage levels and inflammatory status of the lungs during M. tuberculosis infection and fat loss (Fig. 5b to f). The levels of macrophages significantly increased (8-fold), as indicated by F4/80 staining in the lungs of M. tuberculosis-infected mice (Fig. 5b), with no concomitant significant change in TNF-α levels (Fig. 5d and e). The levels of macrophages further increased (*P* ≤ 0.01) in the lungs of infected fat-ablated mice compared to infected fat-unablated mice, which also correlated with significantly reduced (*P* ≤ 0.05) TNF-α levels (Fig. 5d and e). The levels of IFN-γ were higher in the infected fat-unablated mice compared to fat-ablated mice (Fig. 5d and f). Thus, acute fat ablation increases pulmonary levels of macrophages and shifts them toward anti-inflammatory M2 polarization, which is consistent with increased pulmonary M. tuberculosis loads.

### M. tuberculosis infection reduces adipogenesis and increases lipolysis and cell death in visceral fat.

**FIG 6:**
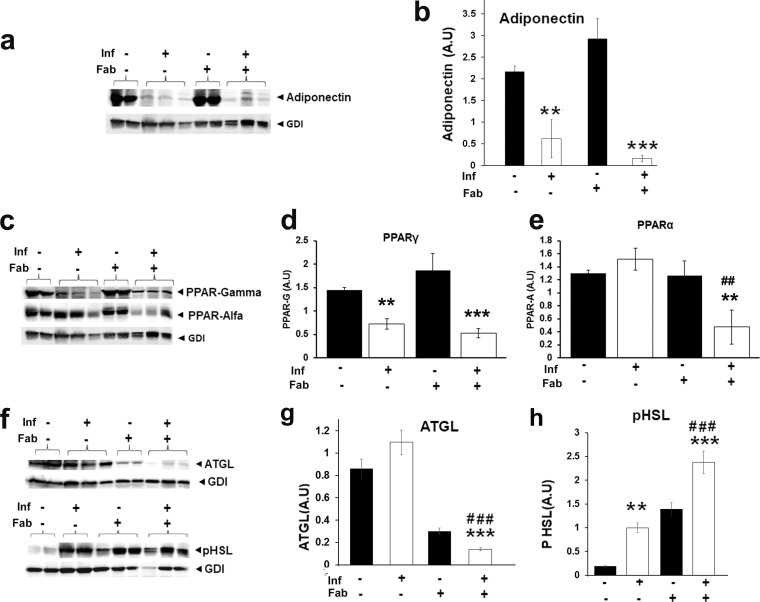
M. tuberculosis infection reduces adipogenesis in the adipose tissue of mice at 30 dpi. (a) Immunoblot analysis of adiponectin in visceral fat tissue lysates of infected and uninfected mice with and without fat ablation. GDI was used as a loading control. (b) Fold changes in the protein levels of adiponectin were normalized to GDI expression and are represented as a bar graph. (c) Immunoblot analysis of PPARs (PPAR-γ and -α) in visceral fat tissue lysates of infected and uninfected mice with and without fat ablation. GDI was used as a loading control. (d) Fold changes in the protein levels of PPAR-γ were normalized to GDI expression and are represented as a bar graph. (e) Fold changes in the protein levels of PPAR-α were normalized to GDI expression and are represented as a bar graph. (f) Immunoblot analysis of lipases (ATGL and p-HSL) in the lysates of visceral fats of M. tuberculosis-infected and uninfected mice with and without fat ablation. GDI was used as a loading control. (g) Fold changes in the protein levels of ATGL were normalized to GDI expression and are represented as a bar graph. (h) Fold changes in the protein levels of p-HSL were normalized to GDI expression and are represented as a bar graph. The error bars represent standard error of the mean. *, *P* ≤ 0.05, **, *P* ≤ 0.01, and ***, *P* ≤ 0.001, compared to uninfected fat-unablated mice. #, *P* ≤ 0.05, ##, *P* ≤ 0.01, and ###, *P* ≤ 0.001, compared to infected untreated mice. Inf, M. tuberculosis infected; Fab, fat ablated.

We evaluated the effect of M. tuberculosis infection on adipose tissue physiology in a murine TB model. Immunoblotting analysis demonstrated a significant decrease in the levels of adipogenic markers, such as adiponectin (3.6-fold; *P* ≤ 0.01) and PPAR-γ (2-fold; *P* ≤ 0.01), in the visceral fat tissue of infected mice compared to uninfected mice at 30 dpi (Fig. 6a and b). Fat ablation further decreased the levels of adiponectin (*P* ≤ 0.001; 10-fold) in the adipose tissue of infected mice compared to uninfected mice. The levels of PPAR-γ in the fat tissues of infected fat-ablated mice compared to infected fat-unablated mice were not significantly different (Fig. 6c and d). While the levels of PPAR-γ decreased during infection, the levels of PPAR-α increased in the infected mice compared to uninfected mice, whereas fat ablation significantly decreased the levels of PPAR-α (3-fold) in the infected mice at 30 dpi (Fig. 6c to e).

The increased levels of PPAR-α suggested M. tuberculosis infection may have caused adipose tissue lipolysis. Thus, we investigated the levels of lipases (ATGL and p-HSL) as markers of lipolysis (Fig. 6). Immunoblot analysis demonstrated a significant increase in the levels of p-HSL (4-fold) in the infected mice compared to uninfected mice (Fig. 6f to h). However, the levels of ATGL and PPAR-α were significantly decreased in the fat tissues of infected fat-ablated mice (5.6- and 3-fold, respectively) compared to uninfected mice and infected fat-unablated mice (*P* ≤ 0.01) (Fig. 6c to h). In addition, the levels of p-HSL were significantly increased (2.5-fold; *P* ≤ 0.001) in the fat tissues of infected fat-ablated mice compared to infected fat-unablated mice (Fig. 6c to h).

### M. tuberculosis infection increases proinflammatory signaling in visceral fat in a murine TB model.

**FIG 7:**
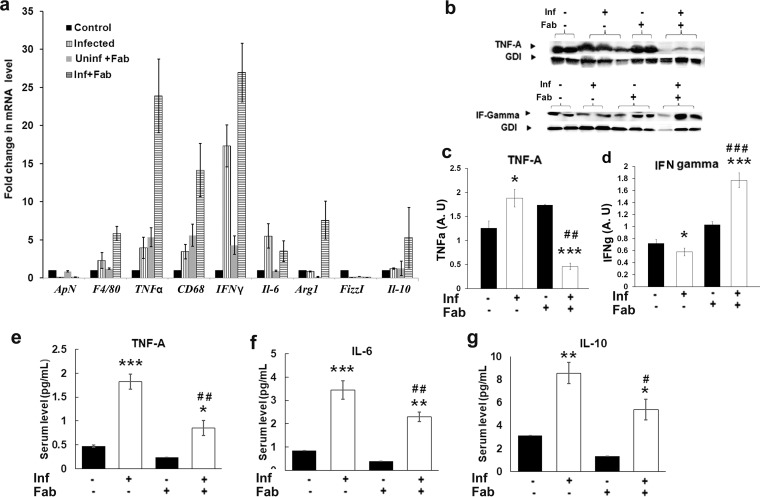
M. tuberculosis infection alters inflammatory molecules in adipose tissue and serum (at 30 dpi). (a) qPCR analysis of mRNA levels of various adipokines and cytokines (adiponectin [ApN], IL-6, TNF-α, IFN-γ, CD68, IL-12, and IL-10) and the markers of M2 macrophages (Arg1 and FizzI) in the visceral adipose tissues of uninfected and infected mice at 30 dpi. Fold changes in the mRNA levels of inflammatory genes were compared to uninfected control mice and normalized to HPRT gene expression and are represented as a bar graph. (b) Immunoblot analysis of proinflammatory cytokines TNF-α and IFN-γ in the lysates of visceral fats of M. tuberculosis-infected and uninfected mice with and without fat ablation. GDI was used as a loading control. (c) Fold changes in the protein levels of TNF-α were normalized to GDI expression and are represented as a bar graph. (d) Fold changes in the protein levels of IFN-γ were normalized to GDI expression and are represented as a bar graph. (e to g) Serum levels of TNF-α, IL-6, and IL-10 in infected and uninfected mice with and without fat ablation measured by the Luminex multiplex analysis system. The error bars represent the standard error of the mean. *, *P* ≤ 0.05, **, *P* ≤ 0.01, and ***, *P* ≤ 0.001, compared to uninfected fat-unablated mice. #, *P* ≤ 0.05, ##, *P* ≤ 0.01, and ###, *P* ≤ 0.001, compared to infected untreated mice. Inf, M. tuberculosis infected; Fab, fat ablated.

The H&E analysis of visceral adipose tissue demonstrated a significant increase in the levels of inflammatory cells during infection, which further increased in the fat-ablated mice at 30 dpi (Fig. 1). qPCR analysis of adipokines and cytokines demonstrated a significant increase in the mRNA levels of macrophages and proinflammatory markers (F4/80, CD68, TNF-α, and IFN-γ) in the adipose tissues of infected mice compared to uninfected mice (Fig. 7a). In contrast, the mRNA levels of anti-inflammatory markers, such as adiponectin (ApN), IL-10, and FizzI, were significantly reduced in the adipose tissues of infected mice (Fig. 7a). qPCR analysis of IL-22 showed no significant difference between the experimental groups compared to the control group. Between the infected fat-ablated and infected fat-unablated mice, the levels of proinflammatory markers further increased (increases in F4/80, CD68, TNF-α, and IFN-γ of 2.2-, 4.5-, 8-, and 2.1-fold, respectively) in infected fat-ablated mice (Fig. 7a). Furthermore, immunoblotting analyses demonstrated significant alterations in the levels of proinflammatory cytokines in the adipose tissues between the infected fat-ablated and -unablated mice compared to uninfected mice (Fig. 7b). The levels of TNF-α significantly increased (1.4-fold; *P* ≤ 0.05) and IFN-γ significantly decreased (1.4-fold; *P* ≤ 0.05) in the fat tissues of infected fat-unablated mice compared to uninfected mice, whereas the levels of TNF-α significantly decreased (2.5-fold; *P* ≤ 0.001) and IFN-γ significantly increased (2.5-fold; *P* ≤ 0.001) in the fat tissues of infected fat-ablated mice compared to uninfected mice (Fig. 7c and d). These data indicate that adipose tissue in infected mice exists in a low-grade proinflammatory state, and an apoptosis-induced acute fat loss selectively increases the levels of IFN-γ. We also analyzed the serum levels of TNF-α, IL-6, and IL-10 using a multiplex assay system. The levels of TNF-α, IL-6, and IL-10 significantly increased (*P* ≤ 0.05) in the serum of infected mice (both fat unablated and fat ablated) compared to uninfected mice (Fig. 7e and f). However, the levels of serum cytokines measured were significantly lower (*P* ≤ 0.05) in the infected fat-ablated mice compared to infected fat-unablated mice. Together these data suggest that elevated cytokine levels in adipose tissue may contribute to the systemic cytokine levels, and an acute loss of fat cells decreases the systemic levels of inflammatory cytokines during infection (Fig. 7e and f).

## DISCUSSION

Wasting is a well-documented manifestation of TB disease and a major determinant of disease severity and outcome (3, 4). This study examines how an acute loss of body fat during M. tuberculosis infection changes the host’s susceptibility to developing active pulmonary disease. Specifically, we used a transgenic fat-amendable murine TB model to examine the effect of fat loss on the pathogenesis of pulmonary TB infection. We also examined how M. tuberculosis infection alters adipose tissue physiology. Our study revealed that M. tuberculosis persists in adipose tissue and increases adipose tissue proinflammatory signaling and that ablation of adipose tissue has drastic consequences for pulmonary pathology, adipokine and cytokine levels, immune cell infiltrations, inflammatory signaling, and bacterial load. These results provide evidence that M. tuberculosis not only infects/persists in adipose tissue, but also regulates pulmonary pathology, which has a significant impact on the severity of disease.

Our data show that even though the site of M. tuberculosis infection and pathogenesis is the lungs in aerosol-infected mice, M. tuberculosis infection directly and indirectly affects adipocyte/fat cell physiology. Acute fat (adipocyte) loss worsens pulmonary pathology in M. tuberculosis-infected mice. Our data further show that acute fat cell loss in adipose tissue, caused by induced fat cell apoptosis, increases pulmonary lipid accumulation (Fig. 1f and g; Fig. S2) and is associated with increased levels of expression of pulmonary adiponectin (Fig. 3). It is possible that lung-accumulated lipid droplets may induce adiponectin overexpression in the alveolar epithelial cells. Although M. tuberculosis infection deregulates adipose tissue physiology and may alter the levels of various adipokines (including reducing levels of adiponectin) at both local and systemic levels, here we focused specifically on adiponectin levels, because adiponectin regulates lipolysis, adipogenesis, and inflammation and is overexpressed in the lungs (14). Clinical studies have demonstrated that adiponectin levels in serum positively correlate with TB severity. Latently infected patients display greater serum adiponectin levels compared to healthy subjects, and active TB patients display higher serum adiponectin levels compared to latent TB infection (LTBI) patients (15, 16). Adiponectin, an anti-inflammatory adipokine, may promote a reduction of TNF-α levels in the lungs in M. tuberculosis-infected mice (Fig. 5), although this change was not significant compared to the level in uninfected mice. However, in the infected fat-ablated mice, the levels of pulmonary adiponectin significantly increased, while levels of TNF-α showed a significant decrease. This suggested that excessive pulmonary adiponectin may promote anti-inflammatory signaling, as indicated by decreased TNF-α levels (Fig. 3 and 5e). In contrast to the fat tissues of infected mice, the lungs of both infected fat-ablated and infected fat-unablated mice showed increased ATGL (Fig. 4 and 6). The levels of lipases (both ATGL and p-HSL) were greater in the lungs of infected fat-ablated mice compared to unablated mice, unlike lipase levels in the adipose tissues of the same groups (Fig. 6). Increased levels of lipases and perilipin in the lungs of infected mice reflected elevated levels of lipolysis and degradation of triglycerides. Therefore, we expected to see—and did indeed see—significantly elevated levels of PPAR-α, a regulator of fatty acid oxidation, in the lungs of infected fat-ablated mice compared to infected fat-unablated mice (Fig. 3). Whether this resulted in decreased IFN-γ levels and whether PPAR-α regulates IFN-γ in the lungs still need to be investigated. These data suggest that normally M. tuberculosis infection results in an increase in lung IFN-γ levels to combat the infection and that the acute infiltration of lipids dampens proinflammatory signaling by reducing both TNF-α and IFN-γ levels. The presence of significantly increased levels of fat-laden foamy macrophages in the lungs of infected fat-ablated mice compared to infected fat-unablated mice supports this hypothesis. In addition, the mRNA levels of M2 macrophage markers (IL-10, arginase 1, and Fizz1) also increased in the lungs of infected fat-ablated mice. Thus, the observed increase in pulmonary bacterial burden in fat-ablated mice may result from the simultaneous decrease of TNF-α and IFN-γ in the lungs (due to inactive macrophages), resulting in an environment that is more permissive of M. tuberculosis replication and survival. However, it is also possible that upon fat ablation the bacteria “hiding” in adipose tissue (lipid droplets) are released and subsequently invade various organs, including the lungs, increasing the pulmonary bacterial burden. All these data suggest that it is important to investigate the pathophysiology of adipose tissue and fat loss in the context of active TB and/or TB reactivation.

Adipose tissue is composed of an adipocyte fraction and a stromal vascular fraction, which contains immune cells, preadipocytes, and endothelial cells (17). It has been shown that immune cells (most notably macrophages, mast cells, neutrophils, and T and B lymphocytes) represent approximately two-thirds of the stromal vascular fraction (18). Thus, adipose tissue not only acts as an active metabolic tissue, but also serves as an immunological organ and contributes to whole-body immune homeostasis (15). We and others have reported that M. tuberculosis infects cultured adipocytes and adipose tissue (5). It is also known that in M. tuberculosis aerosol-infected mice, M. tuberculosis persists in various depots of adipose tissue, such as visceral, subcutaneous, perirenal, and epididymal depots and activates stress-related genes in adipose tissue (7, 9). Here, we demonstrate the presence of M. tuberculosis in visceral fat in an aerosol-infected murine TB model by histological staining, CFU analysis, and qPCR quantitation (Fig. 1 and 2). Our data suggest that bacteria may use adipose tissue as a storage niche during infection and that the loss of adipocytes reduces the bacterial burden in adipose tissue but concomitantly increases bacterial burden in the lungs (Fig. 2).

Although CFU analysis showed a trend, overall bacterial loads in the adipose tissue and lungs were not significantly altered between fat-ablated and fat-unablated groups. One explanation is sampling variability, as we used only a small piece of the lung/adipose tissue for the CFU analysis, which may not represent the bacterial burden in the whole lung. It is also possible that the CFU count may depend on the replication status of the bacteria, which is regulated by nutritional status, and that M. tuberculosis surrounded by lipid-rich adipocyte/lipid droplets may be dormant and less likely to form colonies. However, the qPCR analysis of an M. tuberculosis marker gene (*sigA*) demonstrated increased bacterial load in the lungs of fat-ablated mice compared to fat-unablated mice. Although qPCR analysis of M. tuberculosis genes expressed specifically during active replication or dormancy in the lungs or adipose tissue did not indicate whether acute fat ablation selectively induces either M. tuberculosis replication or dormancy (Fig. S3), overall, the data further supported the conclusion that acute fat loss reduces fat tissue bacterial load but enhances pulmonary bacterial load (Fig. 2).

Adipose tissue plays a regulatory role in lipogenesis and lipolysis. In healthy individuals, adipose tissue physiology is tightly regulated by maintaining an appropriate balance between lipolysis and adipogenesis/lipogenesis, and deregulation of this balance disrupts adipokine secretion and its systemic immuno-metabolic functions. Furthermore, adipokines regulate the activation of localized and systemic immune cells (macrophages and dendritic and T cells) (19). Adiponectin, an anti-inflammatory adipokine, regulates both lipolysis and adipogenesis, depending on insulin and energy levels (13, 14). In addition, both lipolysis and lipogenesis differentially regulate the secretion of adipokines, such as adiponectin, leptin, TNF-α, IL-6, and IL-10, which may further alter the host immune homeostasis and thus influence resistance or susceptibility to M. tuberculosis infection and reactivation. We showed a significant reduction in lipid droplets and an increase in the levels of immune cells in adipose tissue during M. tuberculosis infection (Fig. 1). We hypothesize that during the early acute phase of infection, in order to evade the pulmonary immune burst, many M. tuberculosis*-*infected macrophages move from the lungs to other parts of the body and find a safe and nutritionally rich niche in adipose tissue.

The presence of M. tuberculosis in adipose tissue may trigger proinflammatory signaling via TNF-α produced by resident and infiltrated macrophages. TNF-α binding to adipocytes activates lipolysis and may lead to cell death (apoptosis followed by necrosis) (20). Indeed, our analysis demonstrated a significant increase in the levels of p-HSL and TNF-α in the adipose tissues of M. tuberculosis-infected mice. Increased TNF-α inhibits adipogenesis by regulating the levels of PPAR-γ and adiponectin and induces chronic low-grade lipolysis and a proinflammatory state (21). Adipokine expression in adipose tissue is regulated by adipocyte lipolysis, cell death, and localized inflammatory signaling. An acute loss of adipocytes due to induced apoptosis (our fat ablation model) did not further increase either ATGL or TNF-α protein levels, but did increase p-HSL and IFN-γ levels in the adipose tissue of infected fat-ablated mice compared to infected fat-unablated mice (Fig. 6 and 7).

Increased levels of fatty acid β-oxidation caused by elevated adipocyte lipolysis during M. tuberculosis infection may inactivate other immune cells (such as T helper cells, NK cells, and regulatory T cells), as indicated by a significant reduction in the protein levels of IFN-γ in the adipose tissue of M. tuberculosis-infected mice (Fig. 6 and 7) (22). These data suggest that lipolysis and fatty acid oxidation may be the key factors regulating levels of IFN-γ. PPAR-α is a key regulator of fatty acid oxidation, which is required to catabolize the products of lipolysis. It has been shown that immune cells also express PPAR-α (23). We demonstrated an increase in PPAR-α in the fat tissues of M. tuberculosis*-*infected mice, which significantly decreased in the infected fat-ablated mice (Fig. 6). Given these results, we suggest that adipocyte lipolysis increases fatty acid accumulation in surrounding stromal cellular fractions, thus promoting the expression of PPAR-α, which ultimately results in reduced IFN-γ levels. Thus, M. tuberculosis infection and persistence promote a low-grade proinflammatory environment (as indicated by TNF-α levels) in adipose tissue, causing adipocyte death.

Overall, we have shown that acute loss of adipocytes increased pulmonary levels of lipid droplets, adiponectin, and bacterial burden, which was associated with foamy macrophages and anti-inflammatory signaling. In contrast, a significant loss in the levels of lipid droplets and adiponectin resulted in a low-grade proinflammatory environment in adipose tissue (and systemic levels) in M. tuberculosis-infected mice. Thus, an altered balance between pro- and anti-inflammatory signaling in adipose tissue and the lungs is an important feature of the link between adipose tissue pathophysiology and pulmonary pathology in the context of wasting during TB infection.

### Conclusions.

Our data show that during M. tuberculosis infection of mice, acute body fat loss exacerbates pulmonary pathology, elevates foamy macrophage levels, and increases bacterial load by enhancing lipid accumulation in the lungs and dampening proinflammatory signaling. It is well known that TB patients with active or reactive TB undergo extensive weight loss. Our data suggest that weight loss feeds back on the disease state, with acute body fat loss in M. tuberculosis-infected individuals potentially promoting the progression of active pulmonary TB or TB reactivation in latently infected patients. Therefore, we conclude that adipose tissue and adipokines play a major role in the pathogenesis of activation and reactivation of TB infection.

## MATERIALS AND METHODS

### Animal ethics statement.

All animal experimental protocols were approved by the Institutional Animal Care and Use and Institutional Biosafety Committees of Rutgers University and adhere to the National Research Council guidelines.

### Animal model and experimental design.

The transgenic FAT-ATTAC mice (generous gift from P. Scherer, UT Southwestern Medical Center, Dallas, TX) were bred at the New Jersey Medical School, Rutgers University. Mice were maintained on a 12-h light/12-h dark cycle and housed in groups of two to four, with unlimited access to water and chow (no. 5058; LabDiet). Six- to 8-wk-old male and female FAT-ATTAC (*n* = 12) mice were aerosol infected with M. tuberculosis H37Rv as described earlier (24). Briefly, M. tuberculosis aerosols were generated by a Lovelace nebulizer (In-tox Products, Albuquerque, NM) with a 10-ml bacterial suspension of about 1 × 10^6^ bacilli/ml in saline containing 0.04% Tween 80, and the mice were exposed to the aerosol for 30 min, which results in seeding of approximately 100 colonizing CFU per lung. A separate group of uninfected mice (*n* = 10) was included as controls. At 15 days postinfection (dpi), one set of infected and uninfected mice (*n* = 5/group) were chemically treated to induce fat ablation, as described below. Mice were weighed every 7 days. Mice were sacrificed 30 dpi, and lungs and visceral fat pads (epididymal fat pads) were harvested. Portions of the tissues were homogenized in phosphate-buffered saline-Tween (PBST), and serial dilutions of the homogenates were plated onto Middlebrook 7H10 agar (Difco BD, Sparks, MD) to determine the number of bacterial CFU. Portions of the harvested tissues were fixed in 10% formalin for histological analysis. Portions of tissues were also stored immediately at −80°C for total RNA isolation and protein extraction. The experiment was repeated using the same number of mice. A flowchart describing the experimental design is presented as Fig. S1 in the supplemental material.

10.1128/mBio.02771-18.1 FIG S1 Flowchart of the experimental design. The details of the experimental design, including the number of mice, infection, time points of fat ablation, and sample collection, are presented. Download FIG S1, TIF file, 0.3 MB. Copyright © 2019 Ayyappan et al. 2019 Ayyappan et al. This content is distributed under the terms of the Creative Commons Attribution 4.0 International license.

### Protocol for fat ablation.

AP21087 (Ariad Pharmaceuticals, Cambridge, MA) was administered by intraperitoneal (i.p.) injection at a dose of 0.5 μg/g of body weight. Both uninfected and infected FAT-ATTAC animals (*n* = 5) were injected once daily for 10 days starting at 15 dpi (10, 11).

### Immunoblot analysis.

Protein lysates of the lung and adipose tissue samples were prepared and immunoblot analysis performed as described earlier (25). Adiponectin-specific mouse monoclonal antibody (AB22554, 1:1,000 dilution; Abcam), PPAR-γ-specific rabbit monoclonal antibody (C26H12, 1:1,000 dilution; Cell Signaling), PPAR-α-specific mouse monoclonal antibody (MA1-822, 1:1,000 dilution; Thermo Scientific), phospho-HSL(Ser563)-specific rabbit monoclonal antibody (4139, 1:1,000 dilution; Cell Signaling), ATGL-specific rabbit monoclonal antibody (30A4, 1:1,000 dilution; Cell Signaling), F4/80 antibody-specific mouse monoclonal antibody (sc-377009, 1:500 dilution; Santa Cruz Biotechnology, Inc.), TNF-α-specific rabbit polyclonal antibody (AB6671, 1:2,000 dilution; Abcam), and interferon gamma (IFN-γ)-specific rabbit monoclonal antibody (EPR1108, 1:1,000 dilution; Abcam) were used as primary antibodies. Horseradish peroxidase (HRP)-conjugated goat anti-mouse immunoglobulin (1:2,000 dilution; Thermo Scientific) or HRP-conjugated goat anti-rabbit immunoglobulin (1:2,000 dilution; Thermo Scientific) was used to detect specific protein bands (as explained in the figure legends) using a chemiluminescence system (26). Guanosine nucleotide dissociation inhibitor (GDI) (71-0300, rabbit polyclonal, 1:10,000 dilution; Invitrogen, CA) and a secondary HRP-conjugated goat anti-rabbit antibody (1:2,000 dilution; Amersham Biosciences) were used to normalize protein loading.

### H&E, ZN, and IHC analyses.

Freshly isolated tissues were fixed with phosphate-buffered formalin for a minimum of 48 h and then embedded in paraffin wax. H&E staining was performed, and the images were captured as previously published (27). Four to six images per section of each lung were scored blindly. For each lung sample, histologic evidence of pulmonary pathology was classified in terms of the presence of infiltrated immune cells, lipid droplets, and foamy macrophages and was graded on a 6-point scale ranging from 0 to 5+. ZN staining of the lung sections was performed, and the images were captured (28). Four to six images per section of each lung were quantitated by manually counting the stained bacteria. Immunohistochemical (IHC) analysis was performed on the formalin-fixed lung sections using perilipin-specific antibody (1:250 dilution) followed by HRP-conjugated goat anti-rabbit immunoglobulin (1:1,000 dilution) as previously mentioned (26, 27).

### Serum cytokine analysis.

Serum levels of TNF-α, IL-6, and IL-10 were measured using a Luminex bead-based multiplex assay kit (R&D Systems) according to the manufacturer’s protocol.

### Real-time PCR quantification.

Total host RNA from the lung and adipose tissue was isolated, using the TRIzol reagent (Invitrogen, Carlsbad, CA) as previously reported (24). Isolated RNA was purified by on-column digestion of the contaminating DNA using DNase I. The quality and quantity of the purified RNA were assessed by formaldehyde-agarose gel electrophoresis and a NanoDrop instrument (NanoDrop Products, Wilmington, DE), as previously described (27). RNA was reverse transcribed from 100 ng of total RNA using All-in-One cDNA synthesis supermix (Biotool) according to the manufacturer's protocol. The following primers were used for the amplification of quantitative PCR (qPCR): M. tuberculosis SigA, ESAT6, AGF85A, ACR, DOSR, and host adiponectin (ApN), F4/80, CD68, TNF-α, IFN-γ, arginase 1, FizzI, IL-6, IL-10, IL-22, and HPRT (hypoxanthine-guanine phosphoribosyltransferase) (Table 1). The qPCR was run using Power SYBR green PCR master mix (Thermo Fisher Scientific) following the manufacturer's protocol. To normalize gene expression and to calculate relative mRNA expression, M. tuberculosis *sigA* and mouse *Hprt* levels were measured. For each sample, both the housekeeping and target genes were amplified in triplicate.

**TABLE 1:** Primer sequences used for real-time PCR

Primer	Sequence	
	Forward (5′→3′)	Reverse (3′→5′)
SigA	CTCGACGCTGAACCAGACCT	AGGTCTTCGTGGTCTTCGTC
ESAT6	AGGGTGTCCAGCAAAAATGG	CTGCAGCGCGTTGTTCAG
AGF85A	ATGCAGCTTGTTGACAGGGTT	TCGACGCGACATACCCGT
ACR	GAAGACGAGATGAAAGAGGGG	GTAAGAATGCCCTTGTCGTAGG
DOSR	GAGCTTGACGTCGTAGGTGA	AGAGGTGTAGGACGTGAGGAT
IL-6	CCTCTGGTCTTCTGGAGTACC	ACTCCTTCTGTGACTCCAGC
IL-10	TGGCCCAGAAATCAAGGAGC	CAGCAGACTCAATACACACT
Adiponectin	GCAGAGATGGCACTCCTGGA	CCCTTCAGCTCCTGTCATTCC
F4/80	TTTCCTCGCCTGCTTCTTC	CCCCGTCTCTGTATTCAACC
CD68	ACTTCGGGCCATGTTTCTCT	GCTGGTAGGTTGATTGTCGT
TNF-α	ATGAGCACAGAAAGCATGA	AGTAGACAGAAGAGCGTGGT
IFN-γ	TTCTTCAGCAACAGCAAGGC	TCAGCAGCGACTCCTTTTCC
Arginase 1	GGAATCTGCATGGGCAACCTGTGT	AGGGTCTACGTCTCGCAAGCCA
FizzI	TCCCAGTGAATACTGATGAGA	CCACTCTGGATCTCCCAAGA
IL-22	GCTTGAGGTGTCCAACTTCCAG	ACTCCTCGGAACAGTTTCTCCC
HPRT	GTTGGATCAAGGCCAGACTTTGTT	GAGGGTAGGCTGGCCTATAGGCT

### Statistical analysis.

Statistical analyses were performed using a Student's *t* test, and significant differences were determined as *P* values between <0.05 and <0.001.
