# Supplementary figures and images for: Adipose Tissue Regulates Pulmonary Pathology during TB Infection
Source: mBio. 2019 Apr 16;10(2):e02771-18. doi: 10.1128/mBio.02771-18 (PMC6469978; doi:10.1128/mBio.02771-18)

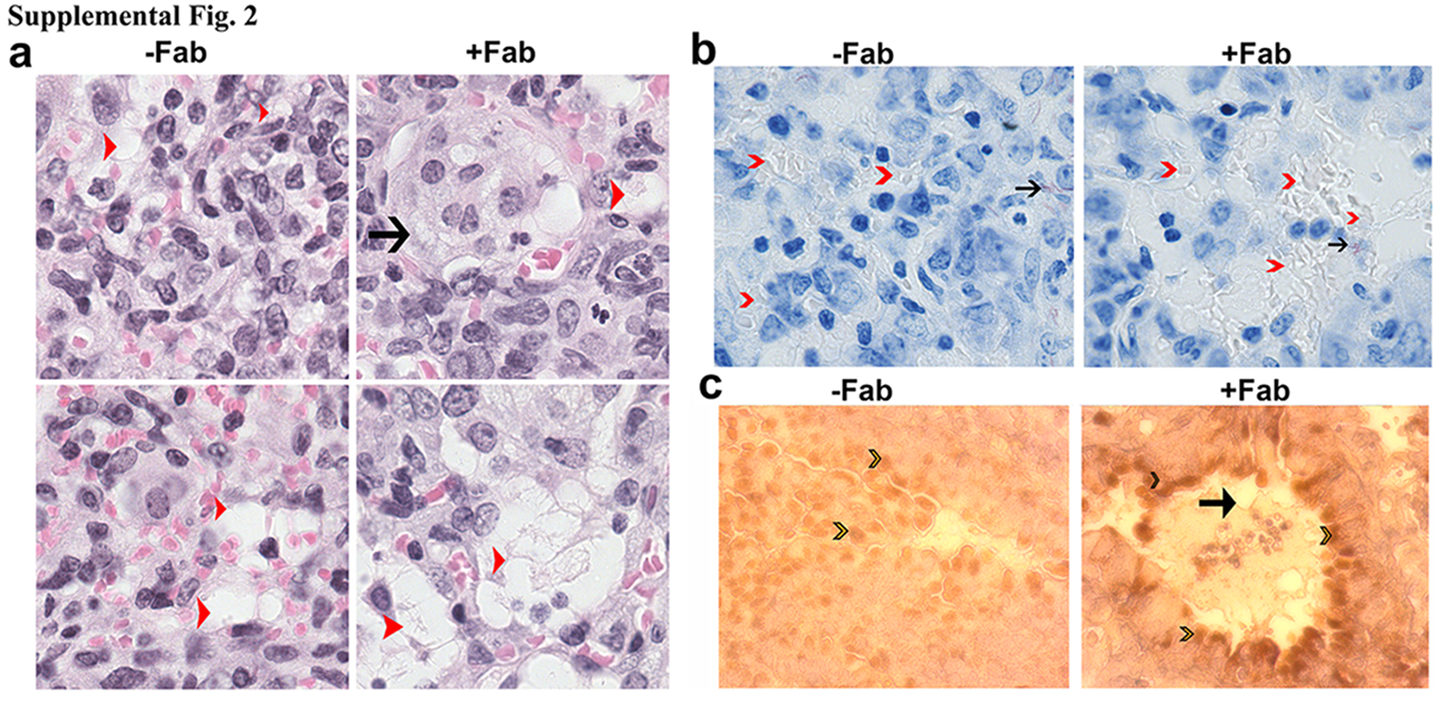

Supplement: FIG S2 [file mBio.02771-18-sf002.tif]

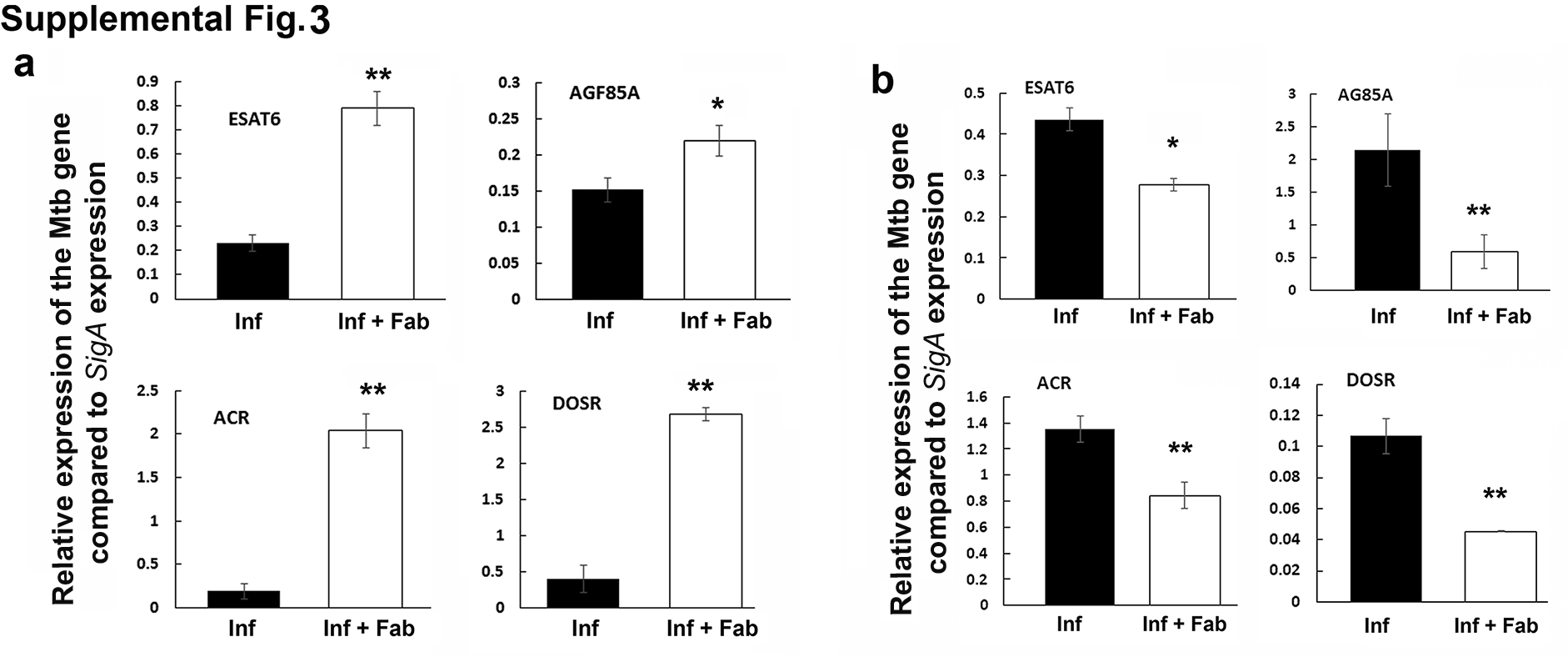

Supplement: FIG S3 [file mBio.02771-18-sf003.tif]

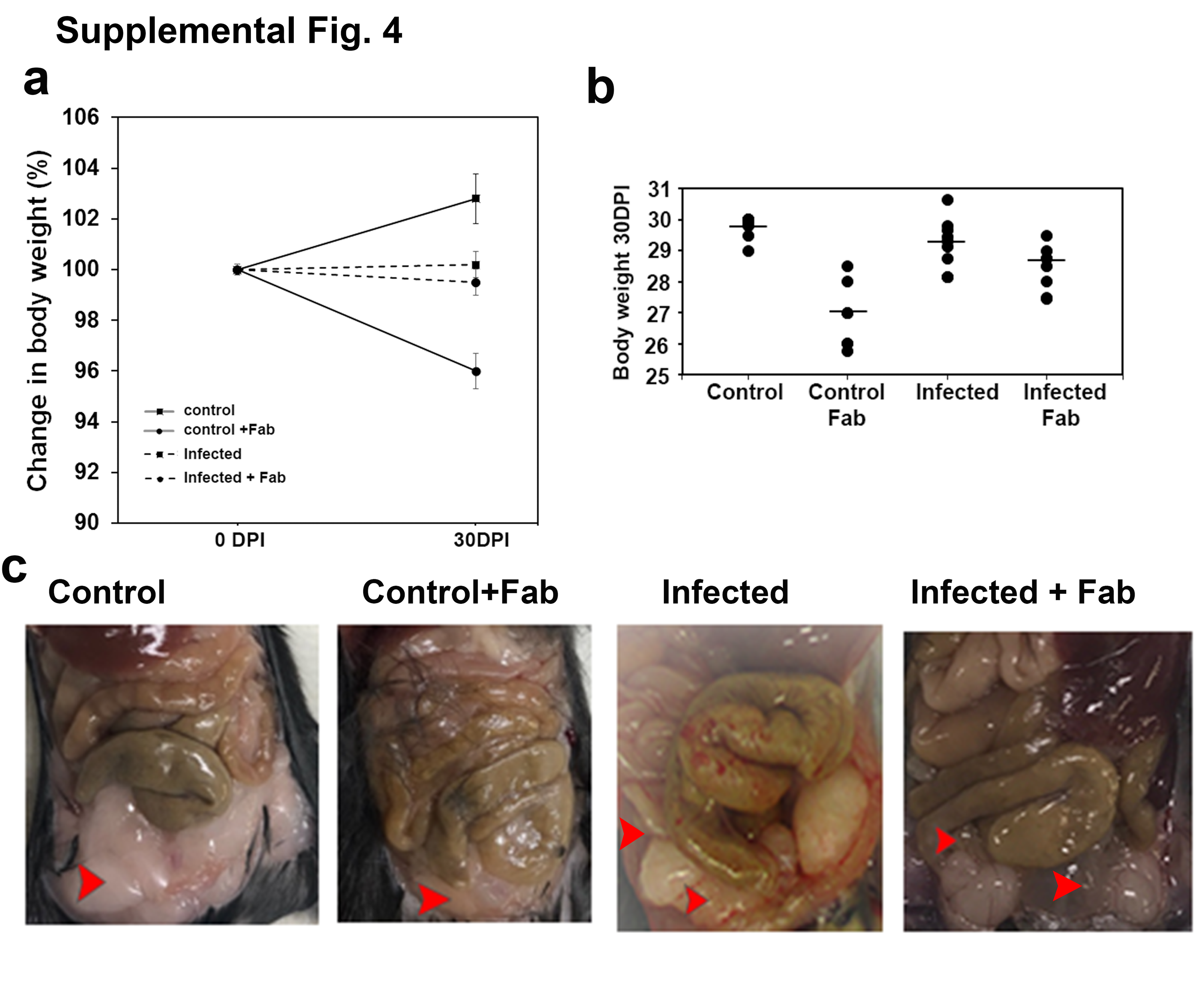

Supplement: FIG S4 [file mBio.02771-18-sf004.tif]

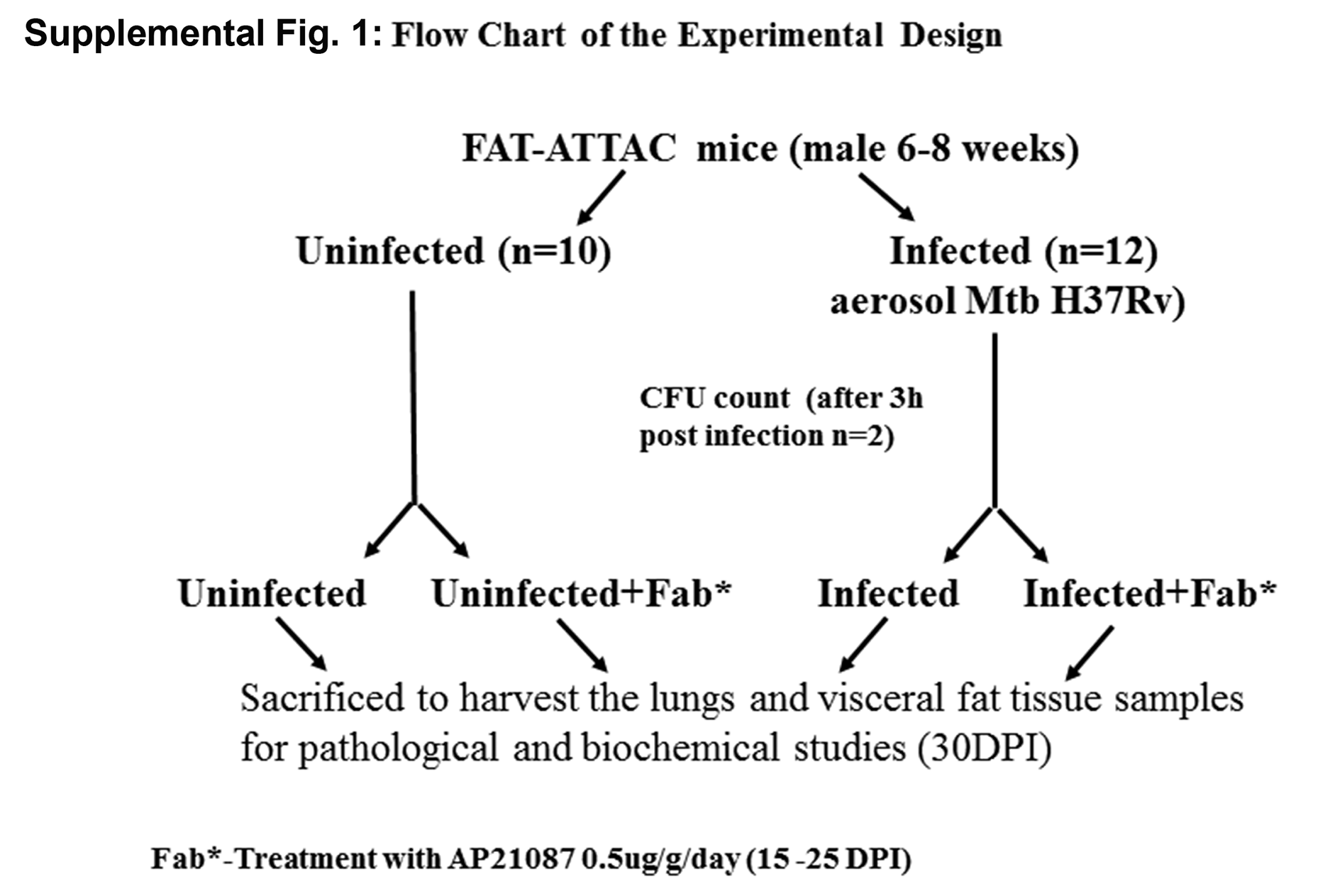

Supplement: FIG S1 [file mBio.02771-18-sf001.tif]
